# The relationship between individual-level deprivation and health-related quality of life

**DOI:** 10.1186/s12955-019-1243-5

**Published:** 2019-11-29

**Authors:** Tahmid Kashem, Fatima Al Sayah, Andrews Tawiah, Arto Ohinmaa, Jeffery A. Johnson

**Affiliations:** 1 School of Public Health, University of Alberta, 2-040 Li Ka Shing Center for Health Research Innovation, Edmonton, AB T6G 2E1 Canada; 2 Faculty of Rehabilitation Medicine, University of Alberta, 3-44 Corbett Hall, Edmonton, AB T6G 2G4 Canada

**Keywords:** EQ-5D-5 L, HRQL, EuroQol, Deprivation, CDI, ODI

## Abstract

**Objective:**

To examine the association between individual-level deprivation and health-related quality of life (HRQL) in the general population.

**Methods:**

Data from a population-based survey in the Canadian province of Alberta were used. Individual-level deprivation was assessed using the Canadian Deprivation Index (CDI) and the Ontario Deprivation Index (ODI). HRQL was assessed using the EQ-5D-5 L. Differences in problems in the EQ-5D-5 L dimensions, index and visual analogue scale (VAS) scores across levels of deprivation were examined. Multivariate logistic and linear regression models adjusted for socio-demographic and other characteristics were used to examine the independent association between deprivation and HRQL.

**Results:**

Of the 6314 respondents, 39% were aged between 18 and 44 years and 38% between 45 and 64 years; 60% were female. Mean EQ-5D-5 L index and VAS scores were 0.85 (standard deviation [SD] 0.14) and 79.6 (SD 17.7), respectively. Almost one-third (30.6%) of respondents reported no problems on all EQ-5D-5 L dimensions. Few participants reported some problems with mobility (23.8%), self-care (6.2%) and usual activities (25.2%), while 59.3 and 35.5% reported some levels of pain/discomfort and anxiety/depression, respectively. Differences between the most and least deprived in reporting problems in EQ-5D-5 L dimensions, index and VAS scores were statistically significant and clinically important. In adjusted regression models for both deprivation indices, the least well-off, compared to the most well-off, had higher likelihood of reporting problems in all EQ-5D-5 L dimensions. Compared to the most well-off, the least well-off had an EQ-5D-5 L index score decrement of 0.18 (*p* < 0.01) and 0.17 (*p* < 0.01) for the CDI and ODI, respectively. Similarly, an inverse association was found between the VAS score and the CDI (β = − 17.3, *p* < 0.01) as well as the ODI (β = − 13.3, *p* < 0.01).

**Conclusion:**

Individual-level deprivation is associated with worse HRQL. Poverty reduction strategies should consider the effects of not only neighbourhood-level deprivation, but also that of individual-level deprivation to improve overall health.

## Introduction

According to World Health Organization’s Commission on Social Determinants of Health-final report, the conditions in which people grow, live and work can directly affect their quality of life [1]. Having a proper education, a decent income, good-quality housing, food security, a sense of social belonging and a sound physical environment allow people to be healthy in all dimensions of health including physical, mental, social, emotional, and spiritual. But inequities in social determinants of health can seriously affect the health by interfering with those basic needs [2].

Deprivation is defined as “a state of observable and demonstrable disadvantage relative to the local community or the wider society or nation to which an individual, family or group belongs.” [3]. Material deprivation is one component of deprivation that includes goods and conveniences necessary for leading a socially acceptable life which meets or rises above the standards of living attained by the majority of the population, such as adequate housing, sports facilities, shops with affordable healthy food and health care facilities [4].

A ‘deprivation index’ is a list of items that has two characteristics [5]. First, the items on this list are widely seen as necessary for a household to have a standard of living above the poverty level. Second, people living in poverty may find some of the items expensive and therefore not have access to them. Deprivation indices are used around the world, with an aim to assess the magnitude and impact of poverty [5]. For example, United Kingdom, Australia and Ireland are reporting deprivation indices as key component of their poverty reduction strategies [5]. In Canada, Quebec and Ontario are using the deprivation indices not only to measure and monitor, but also to assess the progress in reducing poverty [3].

Health related quality of life (HRQL) is a multidimensional construct that includes physical, mental, functional, and social factors determining quality of life [6]. It has the potential to provide a holistic perspective on health status as it goes beyond direct measures of population health and focuses more importantly on the individual’s perceived health status [7, 8]. HRQL’s broad, multi-dimensional, and subjective nature allow it to be used as a comprehensive health indicator in health care and in population health surveys around the world [6]. Furthermore, exploring it in association with deprivation can provide a broader picture on the impact of social inequalities rather than a single health outcome [8, 9].

Neighbourhood-level deprivation, which includes social and economical deprivation, is associated with poor HRQL [7, 8, 10, 11]. Research has shown that lower socio-economic status and neighbourhood deprivation are associated with premature mortality [12], increased incidence of psychosis [13, 14], mental health service use [14], coronary heart disease in adults [15, 16], and behavioural problems in children and adolescents [17, 18]. Studies also suggest that material deprivation plays a significant role in the association between neighbourhood deprivation and poor HRQL [7, 19]. For example, people living in more deprived neighbourhoods suffer from poorer quality of housing [20] and have less access to amenities, such as recreation facilities, health services and food shops [21]. In addition, there is increased use of primary care and hospital service in the deprived areas, presumably because of poorer health [22].

Previous studies have examined the relationship of deprivation and HRQL at the neighbourhood level often using area-based deprivation indicators that link postal code data with census data, assigning individuals’ socioeconomic characteristics of the areas in which they live [7]. However, evidence on the impact of individual-level deprivation on HRQL is lacking. Examining this relationship may better reflect the real-life experiences of deprived individuals and explicate the impact of poverty on HRQL resulting from socioeconomic inequalities [5, 9]. In addition, this could be used at every stage of the health planning process in Canada including the measurement and monitoring of inequalities [23, 24], developing and evaluating the provincial and local services [25], as well as resource allocation [3]. Our aim was to examine the association of individual-level deprivation in the general adult population, using the Canadian Deprivation Index (CDI) and Ontario Deprivation Index (ODI) with HRQL as measured by the EQ-5D-5 L.

## Research design and methods

### Data source

Data from the Alberta Community Health Survey (ACHS) 2015–2016 cycle were used in this analysis [26]. The ACHS is a cross-sectional observational telephone-administered survey of adults in the province of Alberta, Canada. Both cell phone and landline phone numbers were included in the sampling frame, and 7559 adults, representative of the general population of Alberta were recruited. Data on socio-demographic characteristics including age, sex, educational level (not completed high school, completed high school/certificate, university), employment status (employed, out of work/student, unable to work/retired), marital status (married/common-law, widowed/separated/divorced, single/never married), and body mass index (BMI) based on self-reported height and weight were also collected in this survey.

### Measures

#### EQ-5D-5 l

The EQ-5D-5 L is a standardized, generic preference-based measure of HRQL [27]. It consists of two main components, a descriptive system and a visual analogue scale (VAS). The descriptive system consists of five dimensions; mobility, self-care, usual activities, pain/discomfort, and anxiety/depression, each with five levels; 1 “no problems”, 2 “slight problems”, 3 “moderate problems”, 4 “severe problems”, and 5 “extreme problems”, describing 3125 distinct health states, with 11,111 as the best possible and 55,555 as the worst possible health states [28]. The index score was generated based on the Canadian scoring algorithm, which ranges from − 0.148 for the worst (55555) to 0.949 for the best (11111) health states [29]. The index score is anchored at 0 (dead) and 1 (full health), and the minimally important difference for this version of EQ-5D has been reported to be 0.04 [30]. The VAS assesses respondent’s self-rated health on a 20-cm vertical scale, with scores ranging from 0 (worst imaginable health state) to 100 (best imaginable health state).

### Canadian deprivation index (CDI)

The CDI is a measure of material deprivation that consists of three indicators: household education, home ownership, and food security [31]. The household education indicator asks about the highest level of education in a household; responses were categorized into three groups (not completed high school, completed high school/certificate, university). The home ownership indicator captures the number of participants who rented or owned the dwelling; responses were grouped into two categories (owned by a member of this household vs. rented). The food security indicator asks whether respondents worried about running out of food due to lack of money; responses were categorized into three categories (often true, sometimes true or never true). There are three questions on the three indicators and based on the responses, a total score was calculated, with a range from 1 to 5, where “1” was considered as “most well-off”, and “5” as “least well-off”.

### Ontario deprivation index (ODI)

The ODI is a poverty measure developed in association with Daily Bread Food Bank and Caledon Institute of Social Policy in Ontario, Government of Ontario and Statistics Canada [5]. It takes into account 10 items from four deprivation indicators including dietary and health needs (ability to have fresh fruits and vegetables, meat fish or vegetarian equivalent on alternative days, and dental care), clothing and grooming needs (appropriate clothes for job interviews), social inclusion (hobby or leisure activity, ability to buy presents for friends and family once per year, have friends and family over a meal once per month, and being able to get around in your community), and housing (ability to replace or repair broken items, and have a home free of pest). In total, there are 20 questions on the 10 items of four deprivation indicators and the raw score is calculated as the number of deprivation questions answered as “Yes” and having the follow-up reason be due to not being able to afford it. Based on the responses, the total score ranges from 0 to 10 and was categorized into two groups: 0–1 “low” or least deprived, and 2–10 “high” or most deprived.

### Statistical analysis

Participants with complete EQ-5D-5 L data were included in this analysis. For the basic descriptive analysis, the EQ-5D-5 L dimensions were categorized into “level 1= no problem”, “levels 2-3= mild/moderate problem”, “levels 4-5= severe/extreme problem”. For regression analyses, EQ-5D-5 L dimensions were categorized into two groups including “level 1 = no problem” and “level 2-5= having problem”, given the small sample size in levels 3, 4 and 5. Differences in reporting problems in EQ-5D-5 L dimensions, index and VAS scores by levels of deprivation based on both indices were examined using chi-square test, student’s t-test and ANOVA as appropriate. *p* value was set at *p* < 0.05. Effect sizes were calculated as the difference in mean scores of the EQ-5D-5 L index scores for the least well-off and the most well-off on the CDI and ODI scales, divided by the pooled standard deviation. Effect size was interpreted as: 0.2–0.49 small; 0.5–0.79 moderate and > =0.8 as large [32]. The relationship between EQ-5D-5 L dimension, index and VAS scores with the CDI and ODI indices scores was examined using Spearman correlation. Correlation coefficient < 0.2 was considered “absent”, 0.2–0.39 “poor”, 0.40–0.59 “moderate” and > 0.6 “strong”. Multivariable linear regression models and (adjusted for age, sex, marital status and BMI) were conducted to examine the independent association of CDI and ODI with the EQ-5D-5 L index and VAS scores, and multivariable logistic regression models with each of the EQ-5D-5 L dimensions. Given the skewed distributions of both the EQ-5D-5 L index and VAS scores, we used generalized linear models with gamma distribution and re-ran all the models. Results were similar to the linear regression models and therefore the reported results are based on those. Analysis was conducted using STATA version 13.

## Results

### General characteristics of participants

Of the 7559 recruited adult participants, 6314 participants had complete EQ-5D-5 L data and were included in this analysis. Overall, 60% of the respondents were female, 39% were aged between 18 and 44 years, 38% were aged between 45 and 64 years, and 22% were aged between 65 to 75+ years (Table 1). 67% of respondents were married, around 63% completed high school and 58% were employed. The average BMI was 27.52 (SD 5.67) kg/m^2^, with 65% were overweight or obese. Compared to completers, those who did not complete the EQ-5D-5 L (*N* = 1245) were more likely to be female (60.3% vs. 64.5%), with higher proportion of older adults (60.9% vs. 73.3%), be widowed or divorced (16.8% vs. 21.4%), to have lower education (8.8% vs. 11.9%), and to be retired (25.1% vs. 33.5%).

**Table 1:** General characteristics of participants

Characteristic	Overall (*N* = 6314) N (%) or Mean ± SD
Age (Years)	
18–44	2444 (38.7)
45–64	2382 (37.7)
65–75+	1421 (22.5)
Sex- female	3809 (60.3)
Marital Status	
Married/Common-law	4212 (66.7)
Widowed/Separated/ Divorced	1057 (16.7)
Single, never married	1006 (15.9)
Educational level	
High School not completed	553 (8.8)
High School/Certificate	3931 (62.3)
University	1782 (28.2)
Employment status	
Employed	3647 (62.8)
Out of work/Student	703 (12.1)
Retired/Unable to work	1455 (25.1)
Body Mass Index – kg/m^2^	27.52 ± 5.67
Underweight: < 18.5	109 (1.8)
Normal: 18.5–25.0	2013 (33.2)
Overweight: 25.0–30.0	2242 (36.9)
Obese: > 30.0	1702 (28.1)
EQ-5D-5 L	
Mobility	
Level 1	4800 (76.0)
Levels 2–3	1315 (20.8)
Levels 4–5	199 (3.2)
Self-Care	
Level 1	5925 (93.8)
Levels 2–3	361 (5.7)
Levels 4–5	28 (0.4)
Usual Activities	
Level 1	4723 (74.8)
Levels 2–3	1422 (22.5)
Levels 4–5	169 (2.7)
Pain/Discomfort	
Level 1	2571 (40.7)
Levels 2–3	3408 (54.0)
Levels 4–5	335 (5.3)
Anxiety/Depression	
Level 1	4073 (64.5)
Levels 2–3	2089 (33.1)
Levels 4–5	152 (2.4)
Index Score	0.85 ± 0.14
VAS score	79.6 ± 17.7
Canadian Deprivation Index	
1 (Most well-off)	1401 (22.6)
2	3117 (50.4)
3	1175 (19.0)
4	363 (5.7)
5 (Least well-off)	135 (2.2)
Ontario Deprivation Index (Range 0–10)	0.17 ± 0.67
Most well-off (0–1)	5843 (96.1)
Least well-off (2–10)	237 (3.9)

### The relationship of deprivation with EQ-5D-5 L

The mean EQ-5D-5 L index score was 0.85 (SD 0.14), with 30.6% (*n* = 1868) reporting full health (11111), and the mean VAS score was 79.6 (SD 17.7) (Table 1). Most participants (76.2%) reported no problems (level 1) with mobility (76.2%), self-care (93.8%) and usual activities (74.8%). However, 59.3% reported problems (≥ level 2) with pain/discomfort, and 35.5% with anxiety/depression. Mean EQ-5D-5 L index scores were 0.88 (SD 0.10) and 0.85 (SD 0.13) for the most well-off and were 0.71 (SD 0.25) and 0.68 (SD 0.25) for the least well-off, based on CDI and ODI, respectively (Table 2). The mean difference in the index score between the least and most well-off was 0.17 on both CDI and ODI scales, which would be considered a large difference (effect size 1.21). The pattern was similar for VAS scores, where the average scores were 83.1 (SD 14.78) and 80.5 (SD 16.9) for the least deprived, compared to 64.6 (SD 24.2) and 65.9 (SD 23) for the most deprived on the corresponding CDI and ODI index.

**Table 2:** EQ-5D-5 L dimensions, Index and VAS score across different deprivation indices reported as N (%) or mean ± SD

EQ-5D-5 L	Canadian Deprivation Index (CDI)					Ontario Deprivation Index (ODI)	
	1 (Most Well off) (*N* = 1401)	2 (*N* = 3117)	3 (*N* = 1175)	4 (*N* = 363)	5 (Least Well Off) (*N* = 135)	Score 0–1 (*N* = 5843)	Score 2–10 (*N* = 237)
Mobility							
Level 1	1202 (85.8)	2387 (76.6)	830 (70.6)	226 (62.3)	74 (54.8)	4553 (77.9)	117 (49.4)
Levels 2–3	175 (12.5)	666 (21.4)	292 (24.8)	106 (29.2)	45 (33.3)	1133 (19.5)	94 (39.7)
Levels 4–5	24 (1.7)	64 (2.0)	53 (4.5)	31 (8.5)	16 (11.9)	157 (2.6)	26 (10.9)
Self-Care							
Level 1	1363 (97.3)	2948 (94.6)	1081 (92.0)	309 (85.1)	115 (85.2)	5539 (94.8)	190 (80.2)
Levels 2–3	35 (2.5)	160 (5.1)	88 (7.5)	50 (13.8)	17 (12.6)	284 (4.9)	42 (17.7)
Levels 4–5	3 (0.2)	9 (0.3)	6 (0.5)	4 (1.1)	3 (2.2)	20 (0.3)	5 (2.1)
Usual Activities							
Level 1	1159 (82.7)	2351 (75.4)	827 (70.4)	232 (63.9)	68 (50.4)	4476 (76.6)	100 (42.2)
Levels 2–3	223 (15.9)	709 (22.8)	309 (26.3)	100 (27.6)	54 (40.0)	1245 (21.3)	104 (43.8)
Levels 4–5	19 (1.4)	57 (1.8)	39 (3.3)	31 (8.5)	13 (9.6)	122 (2.1)	33 (14.0)
Pain/Discomfort							
Level 1	703 (50.2)	1249 (40.1)	424 (36.1)	113 (31.1)	32 (23.7)	2459 (42.1)	37 (15.6)
Levels 2–3	662 (47.3)	1737 (55.7)	664 (56.5)	201 (55.4)	77 (57.0)	3126 (53.5)	149 (62.9)
Levels 4–5	36 (2.6)	131 (4.2)	87 (7.4)	49 (13.5)	26 (19.3)	258 (4.4)	51 (21.5)
Anxiety/Depression							
Level 1	961 (68.6)	2114 (67.8)	699 (59.5)	173 (47.7)	46 (34.1)	3855 (66.0)	77 (32.5)
Levels 2–3	435 (31.1)	954 (30.6)	441 (37.5)	154 (42.4)	63 (46.7)	1888 (32.3)	119 (50.2)
Levels 4–5	5 (0.4)	49 (1.6)	35 (3.0)	36 (9.9)	26 (19.2)	100 (1.7)	41 (17.3)
Index Score	0.88 ± 0.10	0.86 ± 0.12	0.83 ± 0.16	0.76 ± 0.22	0.71 ± 0.25	0.85 ± 0.13	0.68 ± 0.25
VAS score	83.1 ± 14.8	80.8 ± 16.3	77.1 ± 19.2	71.0 ± 23.2	64.7 ± 24.2	80.5 ± 16.9	66.0 ± 23.0

All differences were statistically significant (*p* < 0.001)

The correlations between the deprivation indices and EQ-5D-5 L dimensions, index and VAS scores were absent to poor (ranging from 0.07 to 0.38). After adjusting for known individual-level characteristics, the least well-off people on the CDI scale were around 6 times (95% CI 3.95, 8.81) more likely to report problems in all dimensions of EQ-5D-5 L compared to the most well-off people (Table 3). Among the most deprived on the CDI scale, the odds of having problems was higher in the mobility dimension (OR = 6.90; 95% CI 4.53, 10.52) compared to the anxiety/depression dimension (OR = 3.32; 95% CI 2.25, 4.90). Similar results were also observed for the least well-off people on the ODI scale. For example, the most deprived people on the ODI scale were approximately 4 times (95% CI 2.97, 6.38) more likely to report problems in all dimensions of EQ-5D-5 L compared to the least deprived. Compared to the most well-off individuals on the CDI index, the least well-off had a decrement of 0.18 points on the index score (*p* < 0.01), while the least well-off on the ODI scale had an index score decrement of 0.17 points (p < 0.01) (Table 4). An inverse association was found between the VAS score and the CDI (β = − 17.3, p < 0.01) as well as the ODI (β = − 13.3, *p* < 0.01).

**Table 3:** Results from multivariable logistic regression analysis of the association of CDI and ODI with EQ-5D-5 L dimensions, adjusted for age, sex, marital status and BMI

Deprivation index	Mobility	Self-Care	Usual Activities	Pain/Discomfort	Anxiety/Depression
	OR (95% CI)	OR (95% CI)	OR (95% CI)	OR (95% CI)	OR (95% CI)
CDI (1 Most well off) – reference					
2	1.61 (1.34, 1.95)	1.80 (1.25, 2.60)	1.40 (1.18, 1.66)	1.37 (1.19, 1.16)	1.01 (0.88, 1.17)
3	2.31 (1.86, 2.88)	2.62 (1.75, 3.9)	1.87 (1.53, 2.29)	1.77 (1.49, 2.10)	1.33 (1.12, 1.58)
4	4.30 (3.20, 5.80)	6.2 (3.92, 9.81)	2.93 (2.21, 3.89)	2.63 (2.01, 3.45)	2.14 (1.67, 2.74)
5 (Least well off)	6.90 (4.53, 10.52)	6.88 (3.77, 12.57)	5.90 (3.95, 8.81)	3.92 (2.53, 6.08)	3.32 (2.25, 4.90)
ODI (Score 0–1) - reference					
Score 2–10	4.17 (3.10, 5.62)	4.47 (3.10, 6.44)	5.21 (3.90, 6.97)	4.35 (2.97, 6.38)	3.61 (2.69, 4.83)

*CDI* Canadian Deprivation Index

*ODI* Ontario Deprivation Index

*BMI* Body Mass Index

All associations were statistically significant at *p* value < 0.05

**Table 4:** Results from multivariable linear regression analysis of the association of CDI and ODI with EQ-5D-5 L index and VAS scores, adjusted for age, sex, marital status and BMI

Deprivation index	Index Score		VAS Score	
	β (SE)	*p* Value	β (SE)	*p* Value
CDI (1 Most well off) – reference				
2	−0.02 (0.004)	< 0.01	−1.6 (0.56)	< 0.01
3	−0.05 (0.006)	< 0.01	−5.1 (0.69)	< 0.01
4	−0.12 (0.008)	< 0.01	−11.4 (1.04)	< 0.01
5 (Least well off)	− 0.18 (0.01)	< 0.01	−17.3 (1.57)	< 0.01
ODI (Score 0–1) – reference				
Score 2–10	−0.17 (0.01)	< 0.01	−13.3 (1.15)	< 0.01

*CDI* Canadian Deprivation Index

*ODI* Ontario Deprivation Index

*BMI* Body Mass Index

*VAS* Visual Analogue Scale

## Discussion

We examined the association between deprivation indices (CDI and ODI) and the EQ-5D-5 L dimensions, index and VAS score, and found that, people with more deprivation were more likely to report problems in all dimensions of the EQ-5D-5 L. The overall patterns were quite consistent; higher CDI and ODI scores were associated with lower EQ-5D-5 L index and VAS scores. These findings suggest that people with more deprivation have lower self-reported health status. Our findings are consistent with other studies that have explored the relationship of deprivation with HRQL, though most of these studies examined neighbourhood-level deprivation [7, 8]. These studies have found that deprivation was associated with more and multiple comorbidities including arthritis, cardiovascular diseases, diabetes and chronic obstructive lung disorders [33–35]. In addition, neighborhood deprivation was also positively associated with depression, alcohol and drugs misuse, anxiety, dyspepsia and pain [36].

Our study focused on individual-level deprivation, however, people living in more deprived neighbourhoods could be affected by their neighbourhood deprivation as well. Research has shown that people living in deprived neighbourhoods are likely to experience lower level of environmental and social quality including high priced low-quality foods, high crime rates, poor housing, toxic environments, limited transportation and lower social cohesion and contacts [33]. These could act as chronic stressors and contribute to the large negative association between neighborhood deprivation and poor health. Another study hypothesized that social group can exert some pressure over and above individual characteristics [37]. For example, smoking behaviour in adolescents comes from the pressure of local peer groups, while unpleasant and unhealthy environment might induce making unhealthy lifestyle choices. On the other hand, it is possible that individual socioeconomic status can drive the association between neighbourhood deprivation and poor health [7]. This is called “social selection” which means people with low socio-economic status tend to cluster together could explain the higher rates of poor health in more deprived neighbourhoods [38].

All of these health burdens could be contributing factors causing the inverse association of individual deprivation with HRQL we observed in this study, even after adjusting for individual characteristics. A further explanation could be unhealthy lifestyles by individuals living in deprived areas, with people being more likely to smoke, less likely to eat sufficient fruits and vegetables or engage in physical activities [20, 39]. Material deprivation including housing and access to amenities could also play an important role in the association between individual deprivation and poor health. For example, living in adverse housing conditions increase the risk of contracting respiratory infections such as asthma [40], chronic illnesses such as cardiovascular disease [41] and mental health problems [42]. In addition, material deprivation may also lead to direct physical risk such as malnutrition and hypothermia which could lead to poor health [43].

This study has a few limitations to note. First, this was a cross-sectional exploration, so the results of this research should be interpreted as associations. As there is the possibility of reverse causation [44], we cannot make any causal inferences about observed relationships. For example, an individual with poor health might have lower income and a different employment status and might be forced to live in a poor housing and unsafe neighbourhood. This, in turn, could change the perception of the health status of that individual. Second, the study participants may not be fully representative of the general Canadian population including overrepresentation of females, married individuals, those who have high school education, and employed individuals; and therefore, the generalizability of the results are somewhat limited. Lastly, our study did not include factors such as neighbourhood (i.e., social cohesion, coziness and social contacts), behavioral (i.e., lifestyle including smoking, alcohol consumptions and illicit drug use) and psychological (i.e., stress or locus of control) characteristics that may have played an important role in the observed associations. Future research should investigate and identify which physical, social, economic, psychological and behavioural factors mediate the association between individual deprivation and HRQL.

### Implications for practice

The findings of this study have implications both in developing and implementing programs and intervention strategies, as well as health services resource allocation. Deprived people are more likely to suffer from multiple comorbidities and have poorer HRQL. Therefor, investing in strategies and interventions aimed at reducing deprivation will not only reduce the socioeconomic inequalities impacting HRQL, but also reduce cost in healthcare delivery and reduce revenue loss.

Overall, deprivation impacts the society, from greater demands on the health care and criminal justice system, to diminished workplace and economic productivity [45]. Many countries around the globe are putting more emphasis on poverty reduction strategies. These strategies or interventions should focus on deprivation at both individual and community level. For example, Alberta’s poverty reduction strategy puts emphasis on income supplementation, appropriate housing, employment, training and skills development as well as place-based initiatives including improving neighborhood crime reduction, peer mentoring and community social events [46]. The findings in our study would aid policy makers in making informed decisions on resource allocation to improve the heavier burden of morbidity in the most deprived areas not only by funding for both primary care teams, social care and community care programs but also through urban planning, housing policies and modifying the food resource environment.

## Conclusion

Individual-level deprivation is associated with worse HRQL. Poverty reduction strategies should consider the effects of not only neighbourhood-level deprivation, but also of individual-level deprivation to improve overall health.

## Data Availability

The datasets used and/or analysed during the current study are available from the corresponding author on reasonable request.

## Abbreviations

ACHS: Alberta Community Health Survey
CDI: Canadian Deprivation Index
EQ-5D-5 L: EuroQol 5 Dimensions 5 Levels
HRQL: Health-Related Quality of Life
ODI: Ontario Deprivation Index
VAS: Visual Analogue Scale
